# Vertebral body versus iliac crest bone marrow as a source of multipotential stromal cells: Comparison of processing techniques, tri-lineage differentiation and application on a scaffold for spine fusion

**DOI:** 10.1371/journal.pone.0197969

**Published:** 2018-05-24

**Authors:** Evangelos M. Fragkakis, Jehan Jomaa El-Jawhari, Robert A. Dunsmuir, Peter A. Millner, Abhay S. Rao, Karen T. Henshaw, Ippokratis Pountos, Elena Jones, Peter V. Giannoudis

**Affiliations:** 1 Leeds Institute of Rheumatic and Musculoskeletal Medicine, University of Leeds, Leeds, United Kingdom; 2 The Leeds Department of Neurosciences, Leeds Teaching Hospitals NHS Trust, Leeds, United Kingdom; 3 Clinical pathology department, Faculty of Medicine, Mansoura University, Mansoura, Egypt; 4 Academic Unit of Trauma and Orthopaedic Surgery, Leeds Teaching Hospitals NHS Trust, Leeds, United Kingdom; The University of Adelaide, AUSTRALIA

## Abstract

The potential use of bone progenitors, multipotential stromal cells (MSCs) helping spine fusion is increasing, but convenient MSC sources and effective processing methods are critical factors yet to be optimised. The aim of this study was to test the effect of bone marrow processing on the MSC abundance and to compare the differentiation capabilities of vertebral body-bone marrow (VB-BM) MSCs versus iliac crest-bone marrow (IC-BM) MSCs. We assessed the effect of the red blood cell lysis (ammonium chloride, AC) and density-gradient centrifugation (Lymphoprep™, LMP), on the extracted VB-BM and IC-BM MSC numbers. The MSC abundance (indicated by colony counts and CD45^low^CD271^high^ cell numbers), phenotype, proliferation and tri-lineage differentiation of VB-BM MSCs were compared with donor-matched IC-BM MSCs. Importantly, the MSC attachment and osteogenesis were examined when VB-BM and IC-BM samples were loaded on a beta-tricalcium phosphate scaffold. In contrast to LMP, using AC yielded more colonies from IC-BM and VB-BM aspirates (p = 0.0019 & *p* = 0.0201 respectively). For IC-BM and VB-BM, the colony counts and CD45^low^CD271^high^ cell numbers were comparable (*p* = 0.5186, *p* = 0.2640 respectively). Furthermore, cultured VB-BM MSCs exhibited the same phenotype, proliferative and adipogenic potential, but a higher osteogenic and chondrogenic capabilities than IC-BM MSCs (*p* = 0.0010 and *p* = 0.0005 for calcium and glycosaminoglycan (GAG) levels, respectively). The gene expression data confirmed higher chondrogenesis for VB-BM MSCs than IC-BM MSCs, but osteogenic gene expression levels were comparable. When loaded on Vitoss™, both MSCs showed a similar degree of attachment and survival, but a better osteogenic ability was detected for VB-BM MSCs as measured by alkaline phosphatase activity (*p* = 0.0386). Collectively, the BM processing using AC had more MSC yield than using LMP. VB-BM MSCs have a comparable phenotype and proliferative capacity, but higher chondrogenesis and osteogenesis with or without using scaffold than donor-matched IC-BM MSCs. Given better accessibility, VB-BM could be an ideal MSC source for spinal bone fusion.

## Introduction

Bone progenitor cells, multipotential stromal cells (MSCs) are increasingly used for the reparative bone therapy. Bone marrow (BM) is the best-studied source for MSCs, being used clinically without or after processing to extract pure culture-expanded MSCs [1]. Spinal deformity (scoliosis, kyphosis), traumatic and degenerative conditions have negative socioeconomic and health impacts with a prevalence of 23% and 14% among adult and children population, respectively [2]. Bone fusion is a conventional method of treating these conditions, however it is not devoid of failures [3]. To promote timely fusion, bone autograft and osteoconductive scaffolds are commonly used. Additionally, the scaffolds can be enriched with BM, usually from the iliac crest (IC), or cultured MSCs to further enhance bone fusion [4–6]. Although a swift biological fusion ensures better preservation of the initial surgical correction and fewer complications, there remains a considerable rate of pseudoarthrosis with subsequent pain and metalwork failure despite the advances in surgical techniques [7–9].

In order to circumvent these complications, a vast amount of work has been undertaken to determine the beneficial synergy between mechanical stability and the use of biological enhancement. Biological stimulation of osteogenesis includes the use of growth factors and MSCs combined with scaffolds and mechanical stability (the diamond concept [10]). However, despite solid scientific evidence [11–13], it appears that the importance of this synergy is often underestimated in the everyday surgical practice.

Although IC remains the gold standard BM-source, its availability could be limited [14]. Technically, vertebral body (VB)-BM harvesting adds virtually no extra time, because vertebral pedicles are approached as a part of the procedure itself and can be extended as far as the metalwork goes, without increasing the complication rate. In recent years, there has been a continuous improvement of our knowledge on BM-MSC characteristics based on the source of origin [15] and the delivery using allogeneic [16] and xenogeneic [17, 18] scaffolds. Although is not determined yet, the optimum combination of these components can represent a promising alternative to autologous bone graft and IC-BM for spinal surgery [19].

Through conducting a literature review from MEDLINE, SCOPUS and EMBASE, seven articles comparing MSCs from IC-BM and VB-BM have been identified between the years 2004–2013 [20–26]. Most of these studies aimed to improve to regenerate the partially or early generated intervertebral disc except Risbud et al. study, which targeted osteogenesis. Although these articles have signalled the importance and clinical potential of VB-BM, they also present certain weaknesses related mainly to the study design and cell-isolation method used. In particular, patient numbers were relatively limited or not clearly stated [20, 23], and BM-MSCs comparisons were made between patients and cadaveric specimens [20, 21]. Finally, in some studies, no donor-matched or paired-sample comparisons were performed [20,21] despite the known age-related variability of MSC counts [27], increasing consecutively the results' heterogeneity.

This study aimed to analyse the effectiveness of VB-BM comprehensively as a source of therapeutic MSCs used for spinal bone fusion. The effect of BM processing techniques; red blood cell lysis versus density gradient centrifugation, on the MSC yield was investigated. The numbers, phenotype, proliferation, and differentiation of VB-BM MSCs particularly those linked to bone formation (the chondrogenesis and osteogenesis) were compared with IC-BM MSCs using donor-matched samples. Furthermore, the attachment and osteogenic differentiation of these MSCs were tested following loading on a beta-tricalcium phosphate (β-TCP) scaffold.

## Materials and methods

### Patients and bone marrow samples

In this study, 25 patients (age range: 13–79 years old, details in Table 1) were recruited under ethics committee approval (06/Q1206/127) from the NRES Committee Yorkshire & The Humber–Leeds East. The participants provided their written informed consent to participate in this study. For the adolescent patients, the consent was obtained from parents or guardians. The patients were undertaking elective spine surgery for correction of adolescent idiopathic scoliosis or degenerative spinal conditions with postero-lateral decompression and fusion. Donor-matched samples of 10-ml of BM aspirates were collected from the posterior iliac crest and 12^th^ thoracic vertebral bodies using known established techniques [23, 28–30]. The bone marrow samples were placed in EDTA containing VACUETTE® blood tubes before the laboratory processing.

**Table 1:** The study patient details.

	Patient ID	Samples	Age (YEARS)	Sex	Clinical phenotype
1	AF 19	IC-BM And VB-BM	13	Female	Adolescent Idiopathic Scoliosis
2	AF 20	IC-BM And VB-BM	15	Female	Adolescent Idiopathic Scoliosis
3	AF 23	IC-BM And VB-BM	13	Female	Adolescent Idiopathic Scoliosis
4	AF 24	IC-BM And VB-BM	14	Female	Adolescent Idiopathic Scoliosis
5	AF 26	IC-BM And VB-BM	14	Male	Adolescent Idiopathic Scoliosis
6	AF 27	IC-BM And VB-BM	16	Male	Adolescent Idiopathic Scoliosis
7	AF 28	IC-BM And VB-BM	14	Male	Adolescent Idiopathic Scoliosis
8	AF 29	IC-BM And VB-BM	15	Male	Adolescent Idiopathic Scoliosis
9	AF 31	IC-BM And VB-BM	15	Female	Adolescent Idiopathic Scoliosis
10	AF 32	IC-BM And VB-BM	16	Male	Adolescent Idiopathic Scoliosis
11	AF 33	IC-BM And VB-BM	16	Female	Adolescent Idiopathic Scoliosis
12	AF 34	IC-BM And VB-BM	14	Female	Adolescent Idiopathic Scoliosis
13	AF 35	IC-BM And VB-BM	15	Female	Adolescent Idiopathic Scoliosis
14	AF 39	IC-BM And VB-BM	15	Female	Adolescent Idiopathic Scoliosis
15	AF 40	IC-BM And VB-BM	16	Female	Adolescent Idiopathic Scoliosis
16	AF 43	IC-BM And VB-BM	16	female	Adolescent Idiopathic Scoliosis
17	AF 48	IC-BM And VB-BM	13	female	Adolescent Idiopathic Scoliosis
18	AF 49	IC-BM And VB-BM	15	female	Adolescent Idiopathic Scoliosis
19	AF 50	IC-BM And VB-BM	17	male	Adolescent Idiopathic Scoliosis
20	AF 51	IC-BM And VB-BM	75	male	Decompression/fusion
21	AF 53	IC-BM And VB-BM	65	male	Decompression/fusion
22	AF 55	IC-BM And VB-BM	79	female	Decompression/fusion
23	AF 57	IC-BM And VB-BM	39	female	Decompression/fusion
24	AF 58	IC-BM And VB-BM	46	Female	Decompression/fusion
25	AF 62	IC-BM And VB-BM	67	Female	Decompression/fusion

The list of the age, gender, sample and clinical phenotype for the patients included in the study.

### Bone marrow processing and MSC culture

All the procedures of BM processing were done under aseptic conditions using biological safety cabinets, Class II. The aspirates of donor-matched IC-BM and VB-BM samples were processed using density gradient centrifugation and red blood cell lysis (5ml of BM for each method). For density gradient centrifugation, BM samples were initially diluted with 1:1 phosphate buffer saline (PBS, Sigma-Aldrich, Dorset, UK) and subsequently layered over a Lymphoprep™ (LMP, Stemcell Technologies, Cambridge, UK). Then the tubes were centrifuged at 800g for 20 minutes with no brake. The layer of mononuclear cells was collected and washed twice with PBS. For red blood cell lysis method, a 0.86% ammonium chloride (AC) solution (Vickers Laboratories, Pudsey, UK) was added to BM samples at 4:1 dilution, followed by 10 minutes’ incubation at 37°C. The BM cells were collected following three washes with PBS. The extracted BM cells by AC and LMP were counted before further use.

Both IC-BM and VB-BM extracted cells were processed to expand MSCs in culture as previously described [31]. The cells were grown in the StemMACS™ MSC Expansion media (Miltenyi Biotec, Surrey, UK) and penicillin/streptomycin (Sigma-Aldrich) at 37°C / 5% CO_2_ culture condition. The cultures were maintained till passage 3 then the plastic-adherent MSCs were detached applying trypsin (Sigma-Aldrich) and used for further processing.

### Colony forming unit-fibroblast assays

The standard colony forming unit-fibroblast (CFU-F) assay was used to count colonies representing MSCs in donor-matched IC-BM and VB-BM samples. For each sample, 2x10^6^ of extracted BM cells were added to the StemMACS MSC Expansion media and seeded in duplicate into 10cm diameter culture dishes (Corning B.V. Life Sciences, Amsterdam, Netherlands). The dishes were cultured for 14 days at 37°C 5% CO_2_ and the media were half-substituted twice weekly. The dishes were then stained using methylene blue dye after fixation with 3.7% formaldehyde [32]. The colonies were manually counted, and the average of two dishes was calculated. The counts were normalised to the volume of 1 ml of BM aspirates.

### Calculation of MSC population-doubling time

As an indicative of MSC proliferation, population-doubling time (PDT) was calculated as previously described [33]. Donor-matched IC-BM and VB-BM MSCs were initially seeded with the density of 1X10^5^ cells/cm^2^ culture flasks until reaching confluence. Subsequently, MSCs were detached from the culture plastic using trypsin and counted. PDT was calculated utilising the equation: PDT = the number of days (D) in the culture divided by the population doubling number. The population doubling number was calculated as log 2 of the MSC numbers at the passage 1 divided by seeding number of MSCs.

### Flowcytometry for counting and phenotype of MSCs

Donor-matched IC-BM and VB-BM samples were processed for flowcytometry to indicate the quantity of bone progenitors in unprocessed BM as described before [34, 35]. In a FACS tube, 100μl of BM was mixed with the MSC positive marker CD271 (Miltenyi Biotec) and haematological cell marker, CD45 (BD Biosciences, Oxford, UK), in addition to dead cell marker, 7-aminoactinomycin D (7AAD, BD Biosciences). The counting beads were used to calculate the absolute numbers of MSCs in the samples according to the manufacturer recommendation. The data were acquired on LSRII 4 laser flowcytometer (BD Biosciences) and were analysed using FACS DIVA software (BD Biosciences).

Flowcytometry was also used to compare the phenotype of culture-expanded IC-BM and VB-BM MSCs. MSCs were examined for the surface expression of the standard MSC markers [36]; CD105 (Miltenyi Biotec), CD73 (BD Biosciences) and CD90 (BIO-RAD, Oxford, UK). Also, hematopoietic lineage markers were included, CD45, CD34, CD14, CD19 and HLA-DR (all from BD Biosciences).

### Osteogenic differentiation assay

For osteogenic differentiation assays, 3x10^4^ culture-expanded MSCs (passage 3) from donor-matched IC-BM and VB-BM samples were cultured in osteogenic media. The osteogenic media was formed of low glucose DMEM (ThermoFisher Scientific Waltham, MA, USA) supplemented with 10% FCS (ThermoFisher Scientific), penicillin and streptomycin (Thermo Fisher Scientific), 100nM dexamethasone, 10mM β-glycerophosphate and 0.05mM ascorbic acid (all from Sigma-Aldrich). After 14 days of culture, the quantification of the calcium level was performed using colorimetric calcium kit (Calcium Liquid, Sentinel Diagnostics, Milan, Italy) as previously described [37]. To extract calcium, cultured MSCs were treated with 1M of HCl solution. The spectrophotometric reading was taken on MULTISCAN EX reader and analysed using Ascent software (Thermo Fisher Scientific). Additionally, the staining for calcium deposition and alkaline phosphatase (ALP) expression was performed using Alizarin Red dye and fast blue RR salt dye respectively (both from Sigma-Aldrich) as used previously [32, 38]. The culture plates were scanned using an Epson 3590 flatbed scanner (Epson Ltd, Hertfordshire, UK).

### Chondrogenic differentiation assay

Donor-matched IC-BM and VB-BM MSCs (expanded for three passages) were seeded at 2.5x10^5^ per conical tube for the chondrogenic assays. Triplicates were used for quantitative measurements of glycosaminoglycan (GAG) levels and duplicates for the GAG staining. As previously described [32], cells were cultured in chondrogenic media, consisting of high glucose DMEM (Thermo Fisher Scientific) supplemented with; l-ascorbic acid-2-phosphate, sodium pyruvate, proline, Bovine serum albumin, penicillin/streptomycin, dexamethasone, insulin-transferrin-selenium (all from Sigma-Aldrich) and TGFβ3 (R&D Systems, Abingdon, UK). Following 21 days of culture, cell pellets were digested in papain solution (100mM Sodium Phosphate Buffer supplemented with 5mM Na_2_EDTA, 10mM l-cysteine and papain, all from Sigma) and the levels of GAG were measured using a Blyscan™ kit (Biocolor Life Sciences, Co Antrim, Ireland) as per manufacturer instructions. For the GAG staining, the cells were treated with 1% toluidine blue (Sigma-Aldrich) then the images for GAG-stained cells were captured using an Eclipse E1000 light microscope (Nikon, Surrey, UK).

### Adipogenic differentiation assay

Adipogenic differentiation assay was performed as previously described [39]. The paired culture-expanded IC-BM and VB-BM MSCs at passage 3 were equally distributed in triplicates with a density of 4x10^4^ per well of 48-wells/plate and cultured in NH AdipoDiff medium (Miltenyi Biotec). Following 21 days of culture, one well was used for the staining of cells with Oil Red O and two wells for the staining of cells with Nile Red/DAPI. The images were captured using an inverted light microscope (IX71 Olympus, Southend-on-Sea, UK) in combination with a fluorescent generator (for Nile Red/DAPI) and an Olympus Digital camera.

### Quantitative real time PCR

The donor-matched VB-BM and IC-BM MSCs were cultured for 1, 2 and 3 weeks in either osteogenic or chondrogenic media as described above. The RNA isolation was performed using Single Cell RNA purification Kit (Geneflow Ltd, Lichfield, UK). The genomic DNA was eliminated from RNA samples using RNase-Free DNase I Kit (Geneflow), and then cDNA was produced using reverse transcription master mix kit (FLUIDIGM, UK). The TaqMan probes (all from Thermo Fisher Scientific) were used to measure the genes of the osteogenic transcription factors; Osteonectin (*SPARC*) and Runt-related transcription factor 1 (*RUNX2*), Osteopontin (*SPP1*), alkaline phosphatase (*ALP*), Collagen type I alpha 2 (*COL1A2*) and Osteocalcin/bone gamma-carboxyglutamic acid-containing protein (*BGLAP*) as well as genes related to chondrogenesis; aggrecan (*ACAN*), Collagen type 2 (*COL2*) and SRY-Box 9 (*SOX9*). The real time PCR assays were run on QuantStudio™ 7 Flex Real-Time PCR System, 384-well (Thermo Fisher Scientific). As published before [40], the data were analysed relative to the housekeeping gene, hypoxanthine-guanine phosphoribosyltrans- ferase (HPRT1), then the fold change of gene in differentiated cells was calculated relative to undifferentiated cells.

### MSC attachment on Vitoss™ scaffold

Unprocessed samples of IC-BM and VB-BM aspirates were seeded onto Vitoss™ (Stryker® UK Limited, Berkshire, UK) as described before [29]. Briefly, 400μl of BM sample was added into 100mm^3^ of Vitoss then incubated at 37°C with gentle rocking for 3 hours to enhance the cell attachment. Each BM sample was used to seed Vitoss in duplicate to examine the MSC attachment and survival using microscopy and flowcytometry respectively. The scaffolds were next rinsed with PBS to remove red blood cells then moved into culture plates in the StemMACS MSC Expansion media and cultured for 2 weeks. After culture, for microscopy, the scaffolds were examined for cell attachment using a SP2 TCS confocal laser scanning microscope (Leica, Buckinghamshire, UK). The DAPI (Thermo Fisher Scientific) and Phalloidin (Sigma-Aldrich) dyes were used to detect cell nuclei and actin, respectively. For flowcytometry-dependent characterisation, the scaffolds were digested using 0.25% collagenase (Stem Cell Technologies). and the released cells were characterised for the surface phenotype of MSCs as previously described [29]. The released cells were stained using CD45, CD90 and CD73 antibodies (BD Biosciences) as well as live/dead markers, CellTrace calcein violet and Aqua-fluorescence reactive dye (Thermo Fisher Scientific), to identify live MSCs.

### MSC osteogenic differentiation on Vitoss™ scaffold

Passage 3 culture-expanded IC-BM and VB-BM MSCs were used to load Vitoss then these scaffolds were cultured for 2 weeks before MSCs being examined for the osteogenic differentiation. The controls were scaffolds cultured in expansion media (the StemMACS MSC Expansion). The scaffolds were lysed in Triton solution and exposed to three cycles of freezing–thawing then centrifuged (16,000g for 1 min) to extract ALP as described previously [41]. The supernatants were collected and used for measuring the ALP activity using the colorimetric kit (BioVision, CA, USA) according to the manufacturer’s instructions. The Optical density was measured at 405nM wavelength using MULTISCAN EX reader, and the ALP activity was calculated using the standard curves. The values of ALP activity were normalised to DNA content (equivalent to cell number) using dsDNA quantitation fluorescence Picogreen kit (Thermo Fisher Scientific).

### Statistical analysis

All statistical analysis and resulting graphs were performed using Prism 6 (GraphPad Software, Inc.). The comparative tests (indicated in the figure legends) for paired groups were chosen depending on whether the data has normal distribution or not. The Gaussian distribution of the data was tested using Shapiro-Wilk and D'Agostino & Pearson omnibus normality tests. The significance level was set at the *p* value of < 0.05.

## Results

### The MSC yield following two processing methods of VB-BM and IC-BM aspirates

**Fig 1:**
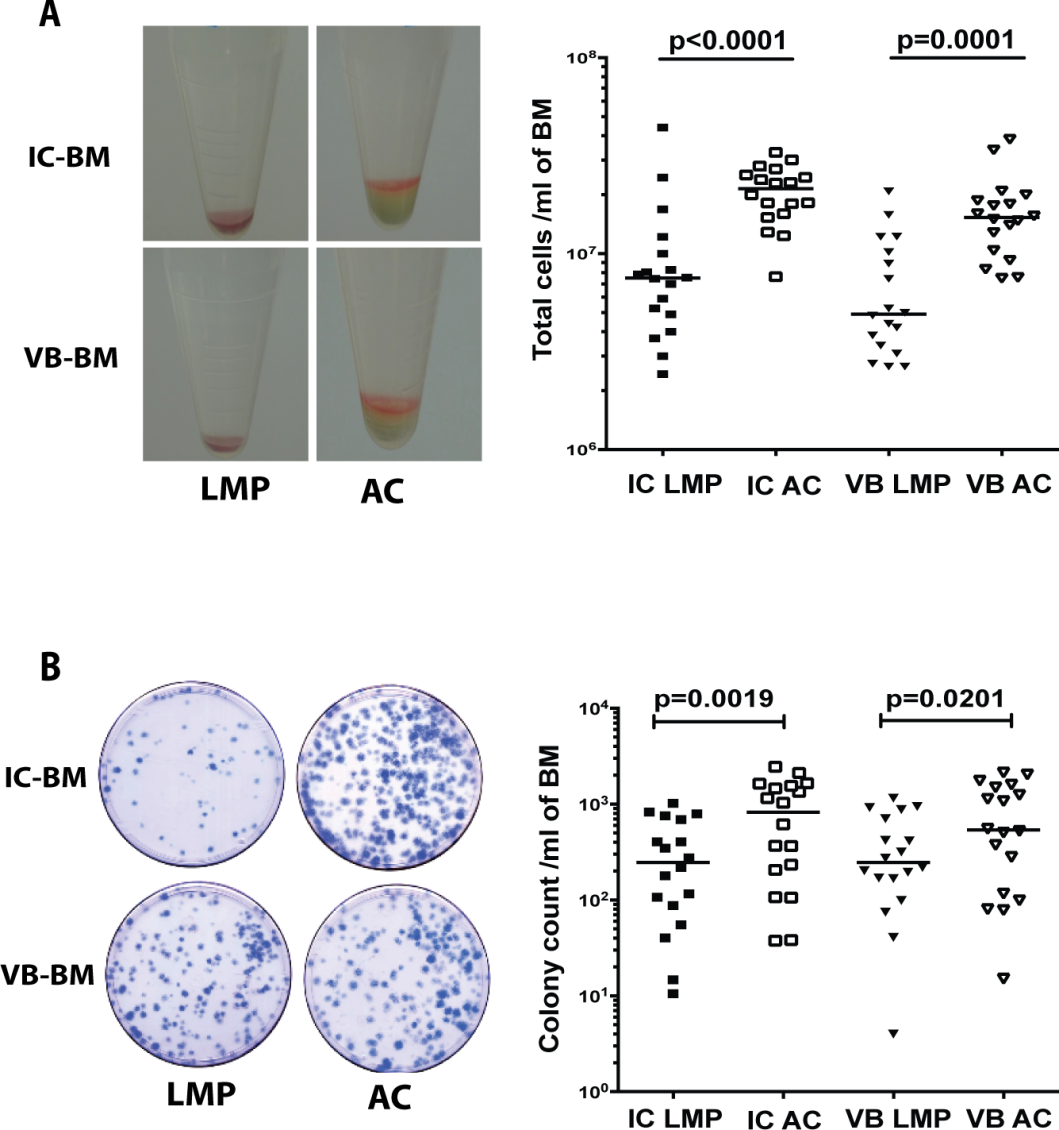
Effect of processing VB-BM and IC-BM aspirates using LMP versus AC. A. A representative example of cell pellet extracted from VB-BM and IC-BM using LMP and AC (left panel). The median of total VB-BM and IC-BM cell numbers extracted using LMP or AC (Friedman with multiple comparisons test, n = 18), (right panel). B. The CFU-F assays; a representative example following 14 days of culture) left panel). The median of total colony numbers for VB-BM and IC-BM samples following processing using LMP or AC (Friedman with multiple comparisons test, n = 18), (right panel). IC; Iliac crest, VB: Vertebral Body, BM: Bone Marrow, LMP: Lymphoprep, AC: Ammonium Chloride.

The samples of VB-BM were aspirated from the vertebral body as illustrated in S1 Fig. The total numbers of yielded cells of IC-BM and VB-BM aspirates were counted to assess the effect of processing method (**Fig 1A, left panel).** The numbers of extracted IC-BM and VB-BM cells consistently similar when processed by the same method (for LMP, p = 0.6065 and AC, p = 0.1556), (**Fig 1A, right panel and S1 File).** However, the total LMP-extracted cell numbers were significantly less (about three-fold) than those extracted using AC for VB-BM (median, LMP: 4.9x10^6^, AC: 15.3x10^6^ cells/ml of BM, *p*<0.0001) and for IC-BM (median, LMP: 7.5x10^6^, AC: 21.46x10^6^ cells/ml of BM, *p* = 0.0001), **(Fig 1A, right panel and S1 File).**

The abundance of VB-BM MSCs and donor-matched IC-BM MSCs was assessed using the colony counts following using LMP and AC **(Fig 1B, left panel).** The MSC colony-forming counts from LMP-processed cells were significantly lower than those from IC-processed cells for VB-BM cells (median, LMP: 247, AC: 540 colonies/ml of BM, *p* = 0.0201;) and AC-BM cells (median, LMP: 247, AC: 824 colonies/ml of BM, *p* = 0.0019), **(Fig 1B, right panel and S1 File)** indicating significant loss of the MSC population using LMP. Of note, no significant difference was detected between the colony counts of donor-matched IC-BM and VB-BM samples extracted using either AC (*p* = 0.5186) or LMP (*p* = 0.9873), **(Fig 1B, right panel and S1 File).** Based on superiority for the MSC yield, all further experiments involving BM processing and comparing VB-BM and IC-BM samples were performed using AC.

### The abundance, proliferation and phenotype of VB-BM MSCs versus IC-BM MSCs

**Fig 2:**
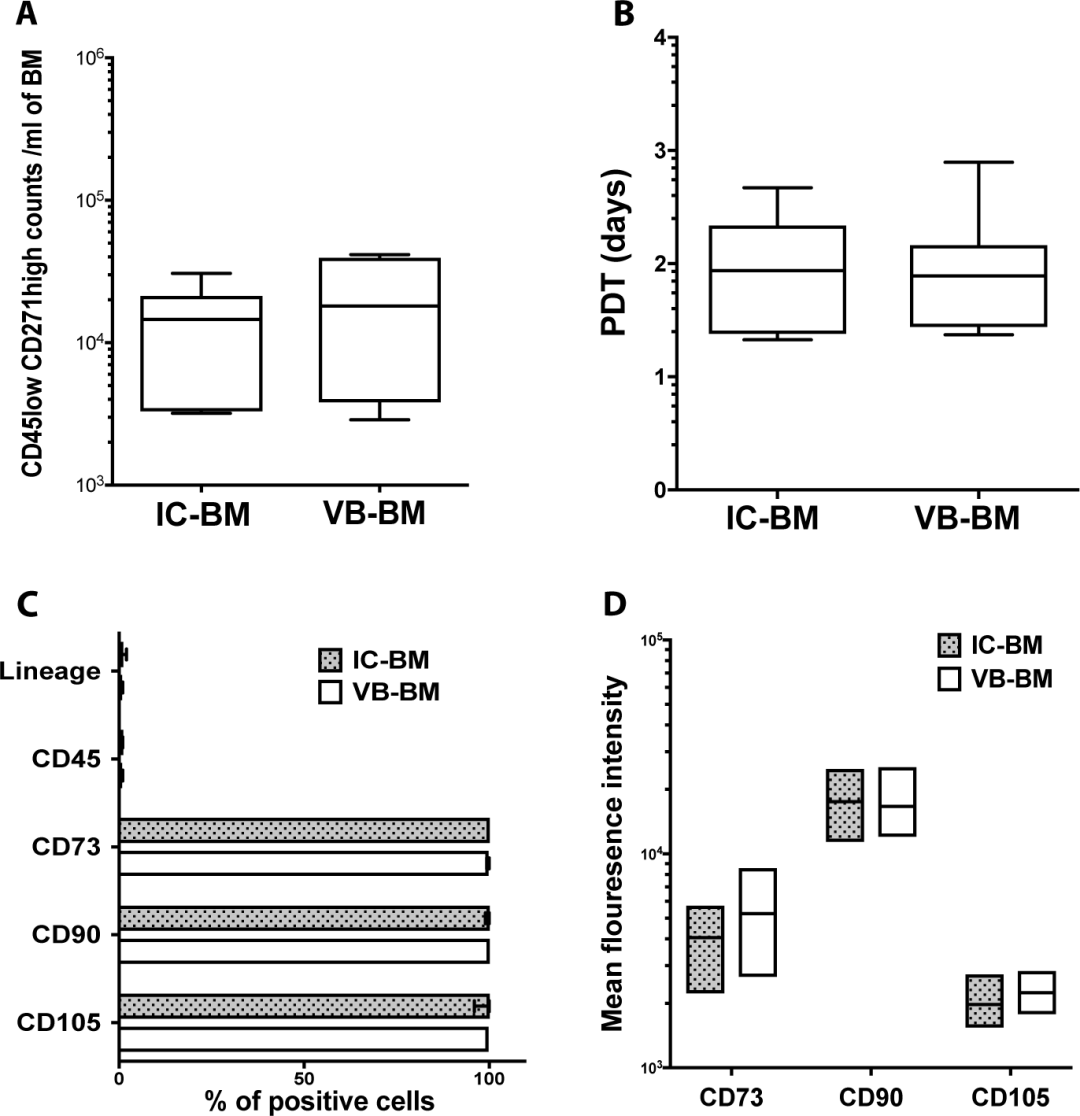
Abundance, proliferation and phenotype of VB-BM versus IC-BM MSCs. A. The numbers of CD45^low^ CD271^high^ cells in unprocessed IC-BM and VB-BM aspirates (means are shown, Paired t-test, n = 6). B. The population doubling time (PDT) for VB-BM versus IC-BM MSCs (means are shown, Paired t-test, n = 7). C. The percentage of culture-expanded VB-BM MSCs versus IC-BM MSCs expressing hematopoietic lineage markers (CD34, CD14, CD19, HLA-DR), CD45, CD73, CD90, and CD105 (n = 3). The means of percentages are shown with bars of the standard error of the mean. D. The mean fluorescence intensity of positive markers expressed on VB-BM and IC-BM MSCs (n = 3).

To confirm the colony number data, the CD45^low^CD271^high^ cell numbers were measured in unprocessed IC-BM and VB-BM aspirates (gating strategy is included in Panel A in S2 Fig) indicating MSC abundance. As expected [42, 43], the data indicated a wide range of donor-related variability for both IC-BM and VB-BM MSCs (2,880–31,700 MSCs/ml of VB-BM and 3,000–41,600 MSCs/ml of IC-BM). Importantly, there was not a significant difference between the CD45^low^CD271^high^ cell numbers in IC-BM and VB-BM aspirates (mean: 14,101 and 20,619 cells respectively, *p* = 0.2640), **(Fig 2A and S2 File).** These data further confirmed a similar abundance of MSCs/ml of donor-matched IC-BM and VB-BM aspirates. We next examined if this similar abundancy of IC-BM and VB-BM MSCs is related to the equal cell proliferation. When expanded in culture, the PDT of donor-matched IC-BM and VB-BM MSCs were approximately two days on the mean, *(p* = 0.9725), **(Fig 2B and S2 File)** indicating a comparable *in vitro* proliferation rate.

The surface phenotype of MSCs for IC-BM and VB-BM cultures was investigated using flowcytometry (gating strategy is included in Panel B in S2 Fig). The data proved the standard MSC phenotype for both IC-BM and VB-BM cultures, showing that only a small percentage of these cultured BM cells (< 1%) expressed CD45 and other hematopoietic lineage markers **(Fig 2C)**. Additionally, IC-BM and VB-BM MSCs were equally positive for CD90 (100%), CD73 (100%) and CD105 (99.8%) **(Fig 2C and S2 File)**. The mean fluorescent intensities of tested positive markers were similar for VB-BM MSCs than IC-BM MSCs **(Fig 2D and S2 File)**. Collectively, these data confirmed the identity of the culture-expanded VB-BM MSCs with a comparable phenotype to that of IC-BM MSCs.

### The tri-lineage differentiation of VB-BM MSCs versus IC-BM MSCs

**Fig 3:**
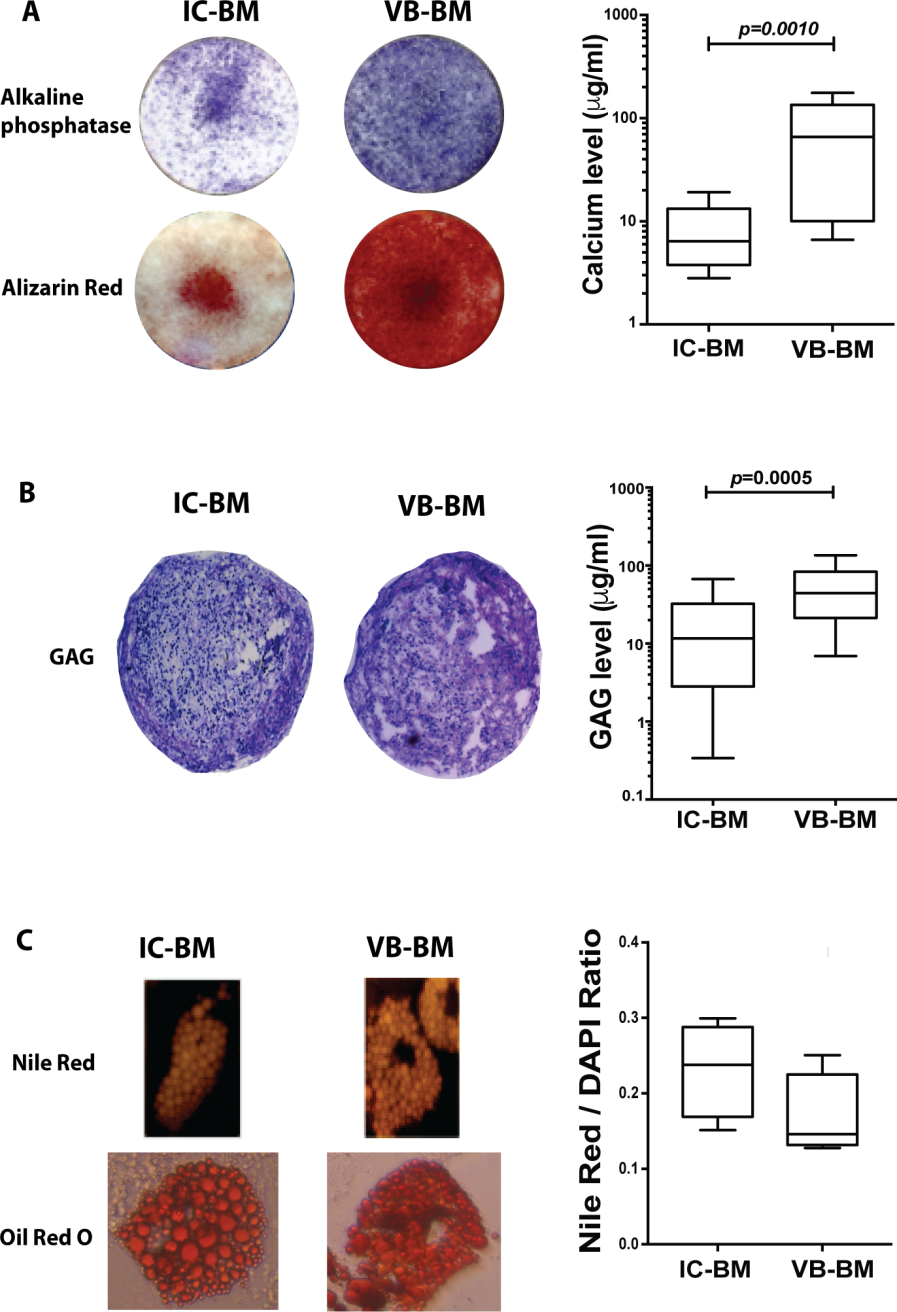
Tri-lineage differentiation capability of VB-BM versus IC-BM MSCs. A. A representative example for testing the osteogenic differentiation of MSCs using alkaline phosphatase staining and alizarin red (left panel). The calcium levels of differentiated VB-BM and IC-BM MSCs (Wilcoxon matched pair rank test, n = 4, each in triplicate, right panel). B. A representative example of the chondrogenic differentiation of MSCs tested by the GAG staining of the cell pellet (left panel). The quantitative GAG levels by IC-BM and VB-BM MSCs (Wilcoxon matched pair rank test, n = 4, each in triplicate, right panel). C. A representative example of the adipogenic differentiation of MSCs using Nile Red and Oil Red O staining (left panel). The quantitative measurement of Nile Red/DAPI ratio for IC-BM and VB-BM MSCs (Paired t-test, n = 4, right panel).

The tri-lineage differentiation was compared between donor-matched culture-expanded IC-BM and VB-BM MSCs. To test the MSC osteogenic differentiation capabilities, the calcium deposition and ALP expression by these MSCs were assessed. The ALP staining suggested a greater level of expression in VB-BM MSCs cultures than in IC-BM MSCs **(Fig 3A, left upper panel)**. Additionally, the Alizarin Red staining showed that VB-BM MSCs could deposit noticeably higher calcium concentrations than that of IC-BM MSCs following osteogenic induction **(Fig 3A, left lower panel)**. The calcium deposition was spectrophotometrically quantified, and it was significantly greater for VB-BM MSCs than IC-BM MSCs (nearly 10-fold, *p* = 0.0010), **(Fig 3A, right panel and S3 File)**. These data demonstrated that VB-BM MSCs could have a higher capability for osteogenic differentiation in 2-dimensional culture in comparison to IC-BM MSCs.

The chondrogenic differentiation was also compared between culture-expanded VB-BM MSCs and IC-BM MSCs via testing the GAG production levels on day 21 post-induction. The pellets formed by both MSCs in chondrogenic media were positively stained for GAG confirming the ability of both VB-BM MSCs and IC-BM MSCs to form cartilage, but greater GAG production was observed for VB-BM MSCs **(Fig 3B, left panel)**. Interestingly, the quantitative GAG measurements confirmed significantly higher levels of VB-BM MSCs, than IC-BM MSCs (nearly 4-fold, *p* = 0.0005), **(Fig 3B, right panel and S3 File)**. These data revealed the superiority of VB-BM MSCs in chondrogenesis.

The adipogenic differentiation of MSCs was examined using the staining of Nile Red and Oil Red O. Both IC-BM and VB-BM MSCs were stained positive for Nile Red and Oil Red O **(Fig 3C, left panel).** The spectrophotometric calculation of Nile Red/DAPI ratio showed no difference between IC-BM and VB-BM MSCs (*p* = 0.2165, **(Fig 3C, right panel and S3 File).** Collectively, these data indicated that VB-BM MSCs were better than IC-BM MSCs regarding osteogenesis and chondrogenesis, but as good in adipogenesis.

### The gene expression for osteogenesis and chondrogenesis of VB-BM MSCs versus IC-BM MSCs

**Fig 4:**
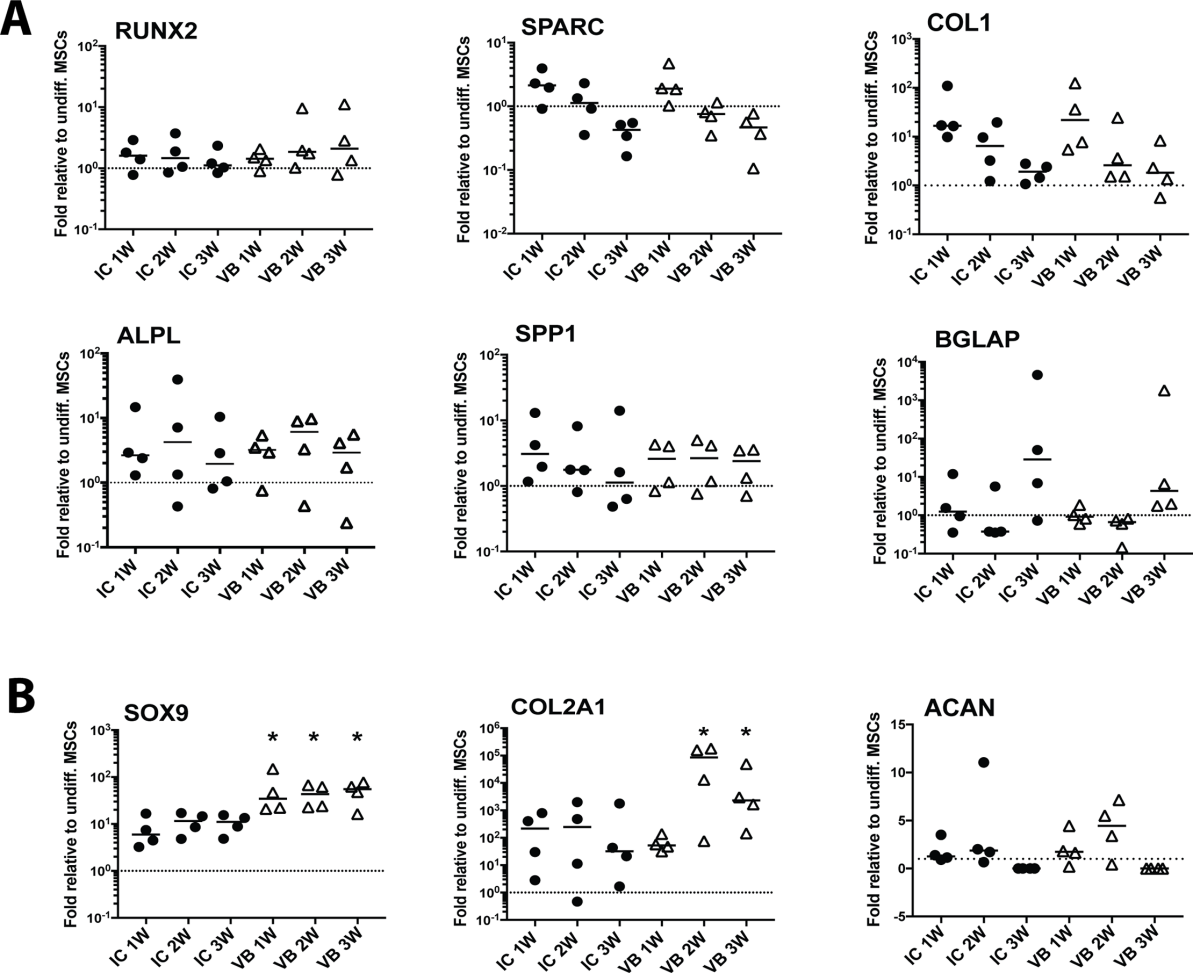
The expression of osteogenic and chondrogenic genes in VB-BM versus IC-BM MSCs. A. The gene expression levels during the osteogenic differentiation of VB-BM and IC-BM MSCs (4 donor-matched pairs) were assessed using TaqMan real time PCR. The gene expressions of samples collected at 1, 2 and 3 weeks of osteogenesis were calculated relative to undifferentiated cells. B. The gene expression levels during the chondrogenic differentiation of VB-BM and IC-BM MSCs (4 donor-matched pairs) were assessed using TaqMan real time PCR. The gene expressions of samples collected at 1, 2 and 3 weeks of chondrogenesis were calculated relative to undifferentiated cells. *: p<0.05. Dotted lines indicate the basal expression level of genes in undifferentiated cells.

The gene expression assays were performed to investigate further the functional differences noted for osteogenic and chondrogenic differentiation between donor-matched IC-BM and VB-BM MSCs. The transcript levels of the osteogenic genes were similarly changed for IC-BM and VB-BM MSCs when measured over time in osteogenic cultures **(Fig 4A and S4 File)**. Relative to undifferentiated MSCs, the gene expression levels for transcription factor RUNX2 were slightly increased with no significant difference between IC-BM and VB-BM MSCs **(Fig 4A, left top)**. The gene expression levels for SPARC were increased after week 1 (median of 2-fold for both) then dropped when measured at week 2 and 3 for both MSCs **(Fig 4A, middle top)**. The collagen 1 gene levels for IC-BM and VB-BM MSCs were markedly increased after one week (median of 16- and 22-fold respectively) then the levels were similarly decreased **(Fig 4A, right top)**. For both IC-BM and VB-BM MSCs, the transcript levels of ALP were increased gradually till week 2 (median of 4- and 6-fold respectively) then the levels dropped at week 3 **(Fig 4A, left bottom)**. The gene levels of osteopontin were similarly increased at week 1 for IC-BM and VB-BM MSCs (median of 3 and 2.6-fold respectively) then minimal changes were noted at week two and three **(Fig 4A, middle bottom)**. In contrast, the osteocalcin gene levels were increased only at week 3 for IC-BM and VB-BM MSCs (median of 28- and 4-fold respectively), but with no statistically significant difference **(Fig 4A, right bottom)**. In total, the patterns of the osteogenic gene expression were similar for IC-BM and VB-BM MSCs.

The chondrogenic genes, aggrecan, collagen type 2 and transcription factor, SOX9 were also tested over three weeks of culture in the chondrogenic milieu (Fig 4B **and S4 File)**. A consistent increase of SOX9 was noted for IC-BM and VB-BM MSCs over week 1, 2 and 3 (IC-BM; 6-, 12-, 11- and VB-BM 34-, 43-, 55-fold respectively). However, the gene levels of SOX9 were significantly higher for VB-BM MSCs relative to IC-BM MSCs at three time-points (*p = 0*.*0446*, *p = 0*.*0069*, and *p = 0*.*0039* respectively). For both MSCs, the median levels of collagen 2 gene levels were increased till week 2 then decreased but stayed higher than undifferentiated cells. The VB-BM MSCs had higher levels of collagen 2 gene than IC-BM MSCs when measured at week 2 and three (*p = 0*.*0248* and *p = 0*.*0078* respectively). The aggrecan gene levels were increased at week 2 for VB-BM MSCs than IC-BM MSCs (median: 2- and 4.5- fold respectively) with no statically significant difference. Together, the chondrogenic gene expression data particularly for SOX9 and collagen 2 confirmed superiority for VB-BM MSCs than IC-BM MSCs.

### The attachment, survival and osteogenic differentiation of VB-BM MSCs on Vitoss™

**Fig 5:**
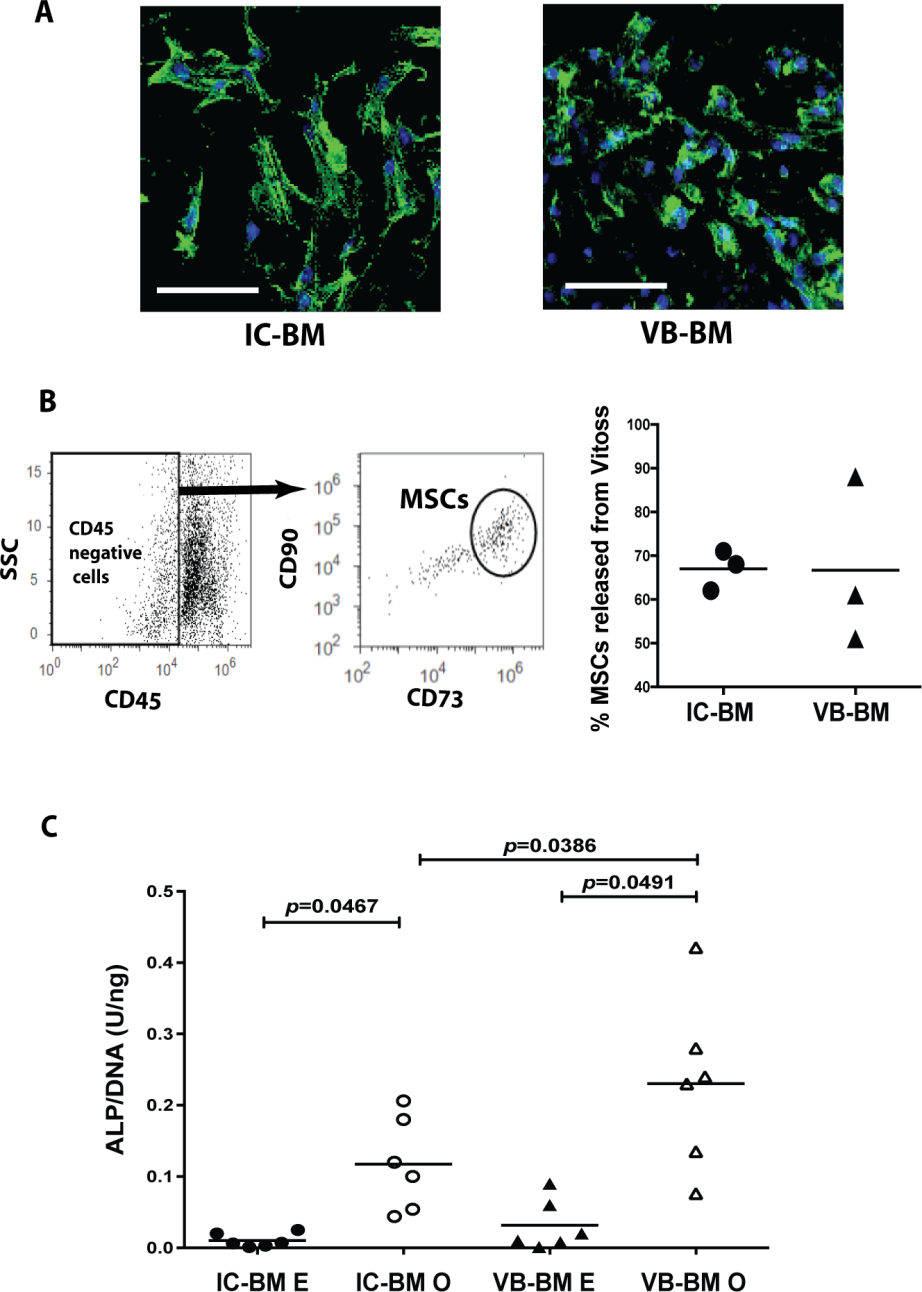
Attachment and osteogenic differentiation of VB-BM MSCs on Vitoss™. A. The cell attachment on Vitoss™ after seeding with aspirates of IC-BM (left panel) and VB-BM (right panel) followed by 2-week culture using confocal microscopy. DAPI staining (blue) was used for cell nuclei and Phalloidin (green) for actin expression. Scale Bar: 50μm. B. The flowcytometry gating strategy for CD45^-^CD90^+^CD73^+^ MSCs released from Vitoss™ following 2-week culture (left panel). The percentage of MSCs out of total CD45^-^ cells for IC-BM MSCs and VB-BM MSCs seeded on Vitoss™ (Paired t-test, n = 3). The mean of the data is shown, right panel. C. The ALP activity levels (normalised to DNA content) for VB-BM MSCs and IC-BM MSCs seeded on Vitoss™ and culture in either osteogenic or expansion media (one-way ANNOVA with Tukey's multiple comparison test, n = 6). The mean of the data is shown. E: expansion media, O: osteogenic media.

The β-TCP scaffold, Vitoss™ was seeded with unprocessed donor-matched IC-BM and VB-BM aspirates then the attachment and the survival of MSCs were compared following 2 weeks of culture. Many cells from both IC-BM and VB-BM were attached to Vitoss™, as shown by confocal images (**Fig 5A).** To quantify MSCs that have survived on the scaffolds during the culture, Vitoss™ was digested, and the released cells were tested for the phenotype of cultured MSCs as CD90^+^CD73^+^CD45^-^ cells **(Fig 5B, left panel)**. The percentages of these IC-BM and VB-BM MSCs were equal (mean, 67% and 67.33% respectively out of CD45^-^ cells, *p* = 0.9836), **(Fig 5B, right panel and S5 File)** indicating a similar ability of both MSCs to attach on Vitoss™.

Vitoss™ was also seeded with culture-expanded MSCs from IC-BM and VB-BM then cultured in osteogenic media to compare the osteogenic capacities of IC-BM MSCs and VB-BM MSCs on scaffolds. The ALP activity level (normalised to cell DNA content) was induced under osteogenic induction, compared to those cultured in expansion medium for both IC-BM MSCs and VB-BM MSCs (*p* = 0.0467 and 0.0491, respectively), (**Fig 5C and S5 File)**. Furthermore, there was a significantly higher level of the ALP activity detected for VB-BM MSCs, than that for IC-BM MSCs (*p* = 0.0386), (**Fig 5C and S5 File)**. Collectively, these results confirmed the advantages of VB-BM MSCs as bone progenitor cells when seeded on the β-TCP scaffolds.

## Discussion

For the evolving field of regenerative bone therapy, the source and the methods of extraction of MSCs are important issues yet to be optimised especially for the spine surgeries. Although VB-BM is a well-known source for hematopoietic stem cell transplantation [44, 45], its use in spinal fusion, as a source of MSCs, is not yet considered as a routine procedure, despite the great benefits of combining MSCs with scaffolds, enhancing the bone formation [11–13]. The combination of β-TCP scaffolds, e.g. Vitoss™ with IC-BM aspirates and/or local bone autograft has been shown to help spinal fusion in pre-clinical and clinical studies [46–48]. However, the outcomes of the clinical trials using these composites could be particularly dependent on the quality of the BM (i.e. the effectiveness of bone progenitor cells). Previous research has reported the differentiation capability of VB-BM MSCs, nevertheless, with lack of consistency in comparison to IC-BM MSCs. Furthermore, the BM harvesting technique and the anatomical site used were not always consistent and a gradient centrifugation method was used throughout all studies, despite the evidence shown for IC-BM, of the considerable loss of MSCs in the precipitate [49, 50] and the higher yield of MSCs with red blood cells lysis method [49, 51]. Here, in a comprehensive donor-matched study, we analysed the effect of the BM processing methods on the quantity of VB-BM and IC-BM MSCs. To our best knowledge, the study here is the first to demonstrate that red blood cell lysis is a more effective method for VB-BM processing with minimal MSC loss compared to the gradient centrifugation. This finding would be of value considering the therapeutic use of the VB-BM samples for the MSC enrichment or culture-expansion of MSCs.

As indicative of bone progenitors in BM samples, the colony counts for VB-BM samples were comparable to that of donor-matched IC-BM samples. In contrast to our findings, the colony counts of VB-BM samples were reported as higher than those of IC-BM samples in two previous studies [23, 24] and lower than IC-BM samples in other two studies [22, 25]. These contradictory data of the colony counts could be related to the variation of the patient cohort and different BM processing/culture methods. Therefore, we used a flowcytometry-based method to confirm that the abundance of the native bone progenitors in VB-BM samples was similar to IC-BM. As we reported recently for IC-BM samples [52], the enumeration of CD45^low^CD271^high^ cells can use as indicative for the quantity of bone progenitors in VB-BM samples.

The data here on cultured VB-BM MSCs showed a comparable standard ISCT marker phenotype and proliferative capacity to IC-BM MSCs in agreement with other reports [20, 21, 24, 26]. Other markers of MSCs such as CD146 has been linked to vascular smooth muscle commitment of BM-MSCs, but no difference in osteogenic, chondrogenic or adipogenic differentiation was noted between CD146^low/negative^ and CD146^high^ MSCs [53]. Additionally, while MSC-like annulus fibrosus cells express contractile Phenotype, the CD146^+^ population of these cells displayed weak osteogenic and chondrogenic differentiation [54]. As our study here focused on the use of MSCs for the bone and cartilage regeneration helping spine fusion, we have not included such marker comparison for VB-BM MSCs in relation to IC-BM MSCs.

Our data, uniquely using quantitative methods, showed that although having similar adipogenic activity, VB-BM MSCs presented a potentially higher chondrogenic differentiation than IC-BM MSCs. In contrast, the capability of VB-BM MSCs to differentiate into cartilage and fat has been demonstrated previously using Safranin O/ Alcian Blue and Oil Red O staining respectively, but without quantification or referral to IC-BM MSCs [21]. Recent studies demonstrated that VB-BM MSCs have a paracrine effect on intervertebral disc cells, enhancing its regeneration reparative process [55, 56]. The *in vitro* co-culture between VB-BM MSCs and disc cells has been shown to induce the disc cell proliferation and collagen synthesis [56]. The greater chondrogenic ability of VB-BM MSCs confirmed here using both functional assays and gene expression data. These data together and given that the chondrogenesis is a step involved in the bone formation, suggested a further potential benefit of using VB-BM MSCs for spinal fusion as well as the disc regeneration purposes.

Our functional data suggested a superior osteogenic differentiation of VB-BM MSCs compared to IC-BM MSCs in agreement with previous studies [21, 26]. Additionally, MSCs extracted from digested VB-bone have been demonstrated to have a higher ALP expression and activity than those extracted from IC-bone [24, 25]. While these studies tested the osteogenesis of VB-BM MSCs using ALP assessment, our study additionally analysed the mineralisation capacity of these MSCs. Importantly, our experiments using Vitoss™ showed that VB-BM MSCs could attach and survive on these scaffolds as efficiently as IC-BM MSCs. As it was difficult to evaluate the mineralisation due to high calcium composition of Vitoss™, ALP activity was only used confirming the greater osteogenic differentiation of VB-BM MSCs on this scaffold relative to IC-BM MSCs. In comparison to functional assays, the gene expression data showed no significant difference of osteogenic markers between VB-BM and IC-BM MSCs. The role of other genes or post-translational factors could explain the higher osteogenic functions of VB-BM MSCs. In addition to the genetic factors, the mechanisms of higher osteogenic/chondrogenic differentiation capabilities of VB-BM MSCs versus IC-BM MSCs can be possibly related to a variation in epigenetic factors regulating the differentiation. The epigenetic changes of MSCs occur sequentially through development to determine lineage-specific differentiation. A distinctive epigenetic signature has been reported explaining the variable differentiation potential between MSCs from BM and adipose tissue [57]. Nevertheless, the findings together highlight the potential value of using VB-BM or cultured VB-BM MSCs seeded on scaffolds to sufficiently enhance the cartilage/bone formation needed for spine fusion.

## Conclusions

Our data showed that the red blood cell lysis is a convenient method for processing VB-BM samples to extract higher yield of MSCs compared to the gradient centrifugation. Despite several studies that have compared IC-BM and VB-BM MSCs, a better comparison of a whole population of MSCs extracted following red blood cell lysis was presented in this study. Beside standard CFU-F assays, flowcytometry was introduced as a quantification assay for MSCs in unprocessed VB-BM aspirates. For each volume unit of BM aspirate, VB-BM MSCs have the same abundance compared to IC-BM MSCs. For the first time, this study revealed a greater chondrogenic ability of VB-BM MSCs compared to IC-BM MSCs. The gene expression profile has been shown to verify this superiority. Uniquely, the advantages of VB-BM MSCs for osteogenesis were quantitatively demonstrated here on both 2- and 3-dimensional culture systems. In particular, we verified the high functional capabilities of VB-BM MSCs when loaded on scaffolds compared to IC-BM MSCs. These biological advantages particularly higher reparative effects together with the easy accessibility, highly suggest the VB-BM aspirates/MSCs as an ideal therapeutic choice for spinal fusion.

## Supporting information

S1 Fig Aspiration of VB BM samples. A. The bone marrow aspirate was collected intraoperatively from VB as shown [left panel) via a 13-gauge bevel-tip introduction needle 5'', Stryker^®^ [in green), inserted in the vertebra by 3-4cm via the pedicle, till the periphery of the vertebral body, then attached to a 10ml syringe. The illustration of the vertebra was reproduced from the Human Anatomy Atlas, Netter 3rd edition, 2006, following explicit permission from Elsevier.
B. For the intraoperative sampling photograph, a Nikon D7000 was used, by the authors.
(TIF)

S2 Fig The gating strategy for characterisation of native and cultured BM MSCs. A. The forward and side scatter (FSC and SSC) of bone marrow cells in bone aspirate with the counting beads are indicated. The BM MSCs were identified as CD45^low^ CD271^high^ cells.
B. The forward and side scatter (FSC and SSC) of culture-expanded BM MSCs. The histograms for the surface markers, hematopoietic lineage markers (CD34, CD14, CD19, HLA-DR), CD45, CD73, CD90, and CD105 are shown.
(TIFF)

S1 File Total cell counts and colony counts. (PDF)

S2 File CD271+ cells, population doubling time and surface markers for MSCs. (PDF)

S3 File Calcium, GAG and Nile red/DAPI. (PDF)

S4 File Gene expression data. (PDF)

S5 File The numbers and ALP levels of MSCs loaded on Vitoss. (PDF)
