# Supplementary figures and images for: Vertebral body versus iliac crest bone marrow as a source of multipotential stromal cells: Comparison of processing techniques, tri-lineage differentiation and application on a scaffold for spine fusion
Source: PLoS One. 2018 May 24;13(5):e0197969. doi: 10.1371/journal.pone.0197969 (PMC5967748; doi:10.1371/journal.pone.0197969)

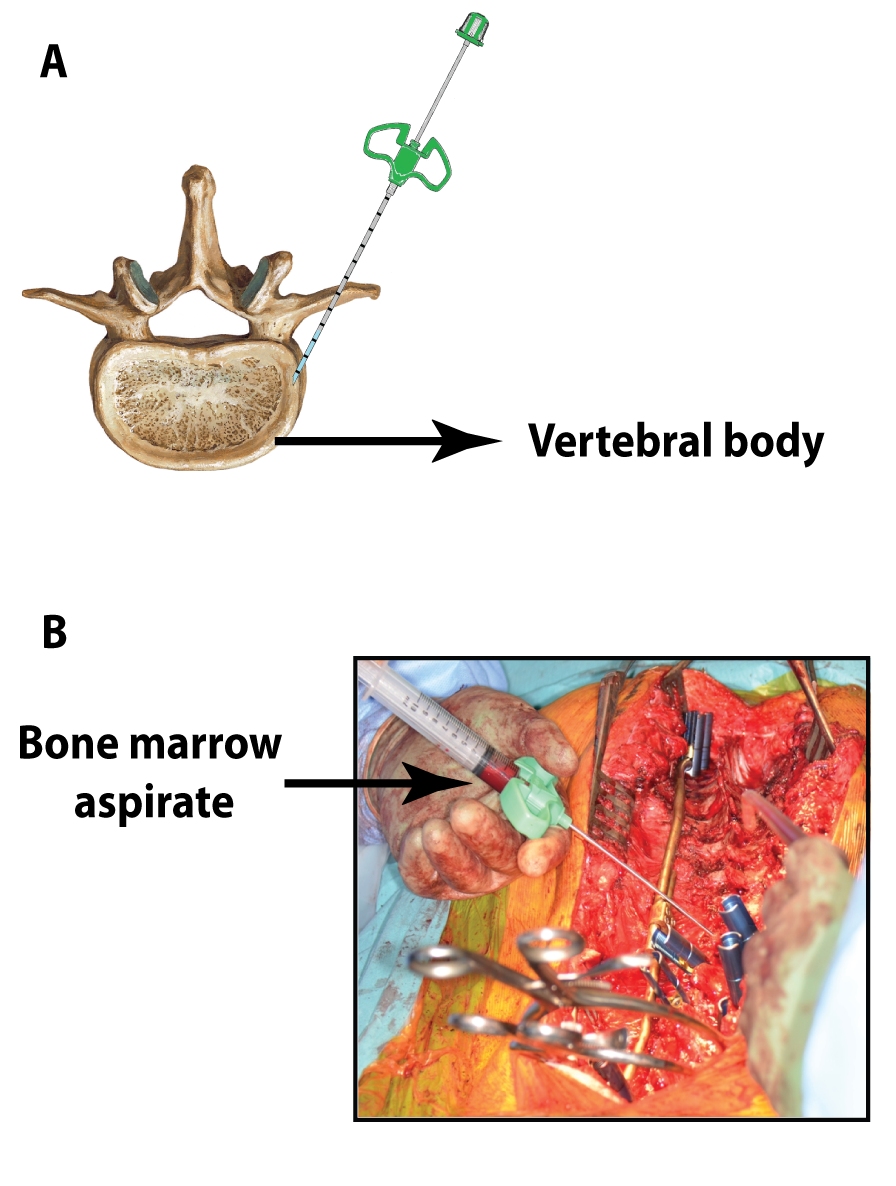

Supplement: S1 Fig — A. The bone marrow aspirate was collected intraoperatively from VB as shown [left panel) via a 13-gauge bevel-tip introduction needle 5'', Stryker® [in green), inserted in the vertebra by 3-4cm via the pedicle, till the periphery of the vertebral body, then attached to a 10ml syringe. The illustration of the vertebra was reproduced from the Human Anatomy Atlas, Netter 3rd edition, 2006, following explicit permission from Elsevier. B. For the intraoperative sampling photograph, a Nikon D7000 was used, by the authors. (TIF) [file pone.0197969.s001.tif]

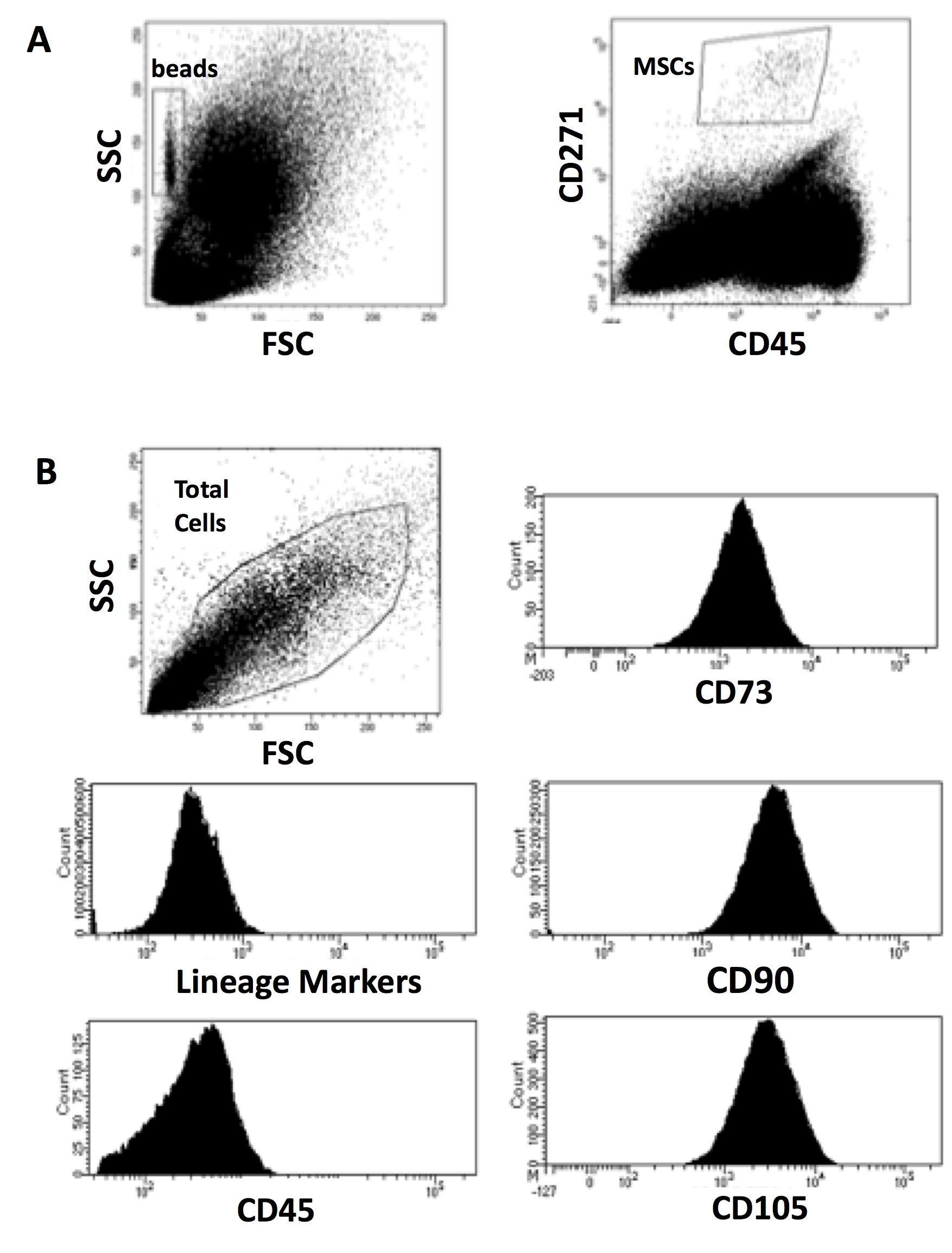

Supplement: S2 Fig — A. The forward and side scatter (FSC and SSC) of bone marrow cells in bone aspirate with the counting beads are indicated. The BM MSCs were identified as CD45low CD271high cells. B. The forward and side scatter (FSC and SSC) of culture-expanded BM MSCs. The histograms for the surface markers, hematopoietic lineage markers (CD34, CD14, CD19, HLA-DR), CD45, CD73, CD90, and CD105 are shown. (TIFF) [file pone.0197969.s002.tiff]
