# Supplementary material for: Vertebral body versus iliac crest bone marrow as a source of multipotential stromal cells: Comparison of processing techniques, tri-lineage differentiation and application on a scaffold for spine fusion
Source: PLoS One. 2018 May 24;13(5):e0197969. doi: 10.1371/journal.pone.0197969 (PMC5967748; doi:10.1371/journal.pone.0197969)
Supplement: S1 File — (PDF) [file pone.0197969.s003.pdf]

TOTAL CELL count/ml of BM

	IC LMP	IC AC	VB LMP	VB AC
sample 1	2430000	12360000	2660000	9250000
sample 2	8050000	12880000	8900000	17600000
sample 3	24470000	32890000	3830000	8330000
sample 4	7830000	23100000	12250000	18670000
sample 5	5890000	18200000	10200000	17900000
sample 6	4900000	18180000	2660000	7560000
sample 7	8270001	27080000	12220000	20890000
sample 8	4000000	16000000	4400000	10400000
sample 9	16840000	25260000	20820000	33870000
sample 10	3000000	18000000	3100000	20000000
sample 11	10000000	20000000	5000000	15600000
sample 12	7000000	15300000	3400000	15000000
sample 13	3700000	23900000	2750000	14000000
sample 14	7460000	7630000	4200000	7500000
sample 15	7550000	22910000	7450000	16000000
sample 16	12200000	24500000	4840000	12840000
sample 17	5260000	30110000	5260000	14740000
sample 18	44000000	28000000	15790000	38360000
Median	7505000	21455000	4920000	15300000
Mean	10158333	20905556	7207222	16583889

colony count/ml of BM

	IC LMP	IC AC	VB LMP	VB AC
sample 1	218.7	367.71	167.54	99.44
sample 2	40.25	206.08	413.85	281.6
sample 3	55.06	106.89	74.69	116.62
sample 4	179.44	1647.39	170.14	525.09
sample 5	792.21	1638	706.35	505.68
sample 6	14.7	37.62	4.01	15.12
sample 7	106.82	365.58	40.73	78.34
sample 8	404.41	38.15	219.19	375
sample 9	348.16	1159.88	420.8	554.27
sample 10	828.57	1460	1159.4	2137.2
sample 11	87.57	233.89	923.89	2041.74
sample 12	10.54	106.22	317.17	1600.67
sample 13	115.99	615.83	877.2	1076.47
sample 14	403.64	2120	98.75	1497.6
sample 15	753.11	2451.37	946.15	1760
sample 16	275.25	1033.1	195.3	1135
sample 17	1021.75	1341.38	274.27	80.25
sample 18	691.43	1550	202.5	1246.7
Median	247	824.5	246.7	539.7
Mean	352.6	915.5	400.7	840.4
