# Supplementary material for: Vertebral body versus iliac crest bone marrow as a source of multipotential stromal cells: Comparison of processing techniques, tri-lineage differentiation and application on a scaffold for spine fusion
Source: PLoS One. 2018 May 24;13(5):e0197969. doi: 10.1371/journal.pone.0197969 (PMC5967748; doi:10.1371/journal.pone.0197969)
Supplement: S2 File — (PDF) [file pone.0197969.s004.pdf]

The numbers of CD45^{low} CD271^{high} cells

	IC-BM	VB-BM
sample 1	3328	4128
sample 2	3200	2880
sample 3	15680	4800
sample 4	30720	41600
sample 5	18240	31360
sample 6	13440	38944
mean	14101	20619

The percentage of cells expressing surface markers

	IC-BM Lilineage	VB-BM lineage	IC-BM CD45	VB-BM CD45	IC-BM CD73	VB-BM CD73	IC-BM CD90	VB-BM CD90	IC-BM CD105	VB-BM CD105
SAMPLE 1	2	1	0	1	100	100	100	100	100	100
SAMPLE 2	0	1	1	0	100	99	99	100	96	99
SAMPLE 3	1	0	1	1	100	100	100	100	100	100
Mean	1	0.6667	0.6667	0.6667	100	99.67	99.67	100	98.67	99.67

The mean fluorescence intensity of MSC markers

	IC-BM CD7[illegible]	VB-BM CD73	[illegible]-BM CD9[illegible]	[illegible]B-BM CD9[illegible]	[illegible]-BM CD1[illegible]	[illegible]B-BM CD105
SAMPLE 1	5706	2667	24890	12558	1650	2824
SAMPLE 2	4246	4555	16380	12041	2730	2126
SAMPLE 3	2231	8556	11396	25354	1544	1784
Mean	4061	5259	17555	16651	1975	2245

The population doubling time (PDT)

	IC-BM	VB-BM
sample 1	1.327158	1.435462
sample 2	1.372612	1.371511
sample 3	2.342332	2.168318
sample 4	2.112847	2.896773
sample 5	2.671289	1.714735
sample 6	1.937956	1.892838
sample 7	1.751566	1.986504
Mean	1.931	1.924
