# Supplementary material for: Vertebral body versus iliac crest bone marrow as a source of multipotential stromal cells: Comparison of processing techniques, tri-lineage differentiation and application on a scaffold for spine fusion
Source: PLoS One. 2018 May 24;13(5):e0197969. doi: 10.1371/journal.pone.0197969 (PMC5967748; doi:10.1371/journal.pone.0197969)
Supplement: S3 File — (PDF) [file pone.0197969.s005.pdf]

calcium levels

	IC-BM	VB-BM
smaple 1 a	7.947288	19.24932
smaple 1 b	8.712529	14.48128
smaple 1 c	5.239508	16.8653
sample 2 a	2.815865	118.3905
sample 2 b	3.335116	139.9761
sample 2 c	3.162033	176.3633
sample 3 a	7.354691	6.875583
sample 3 b	5.498147	6.636028
sample 3 c	5.078928	8.552459
sample 4 a	19.15272	112.3685
sample 4 b	14.72097	146.3901
sample 4 c	18.25439	115.8002
Median	6.426	65.81

GAG levels

	IC-BM	VB-BM
smaple 1 a	41.1145	135.406
smaple 1 b	67.1084	90.04417
smaple 1 c	36.7822	106.3541
sample 2 a	9.514103	63.03093
sample 2 b	10.53347	60.73737
sample 2 c	15.88515	62.52123
sample 3 a	0.339792	19.45295
sample 3 b	0.594629	13.33673
sample 3 c	0.594629	6.902613
sample 4 a	19.1981	27.86272
sample 4 b	12.82705	27.0982
sample 4 c	10.53347	27.7161
Median	11.68	44.3

Nile Red/DAPI ratio

	IC-BM	VB-BM
sample 1	0.2219242	0.250416
sample 2	0.2993495	0.143685
sample 3	0.2535248	0.1478769
sample 4	0.1512501	0.1273422
mean	0.2315	0.1673
Median	0.2377	0.1458
