# Supplementary material for: Vertebral body versus iliac crest bone marrow as a source of multipotential stromal cells: Comparison of processing techniques, tri-lineage differentiation and application on a scaffold for spine fusion
Source: PLoS One. 2018 May 24;13(5):e0197969. doi: 10.1371/journal.pone.0197969 (PMC5967748; doi:10.1371/journal.pone.0197969)
Supplement: S4 File — (PDF) [file pone.0197969.s006.pdf]

FOLD RELATIVE TO UNDIFFERENTIATED MSCs

	IC 1W	IC 2W	IC 3W	VB 1W	VB 2W	VB 3W
RUNX1						
smaple 1 a	1.3993	3.721853	1.024765	0.896558	9.58118	11.15699
smaple 1 b	1.3993	3.721853	1.024765	0.896558	9.58118	11.15699
sample 2 a	1.811277	0.848937	1.203285	1.354532	1.949657	1.350952
sample 2 b	1.811277	0.848937	1.203285	1.354532	1.949657	1.350952
sample 3 a	0.77869	1.052775	0.839116	1.497921	1.755539	0.781434
sample 3 b	0.77869	1.052775	0.839116	1.497921	1.755539	0.781434
sample 4 a	2.898248	1.879681	2.329512	2.063146	1.022962	2.834673
sample 4 b	2.898248	1.879681	2.329512	2.063146	1.022962	2.834673
SPARC						
smaple 1 a	3.946787	2.299416	0.511379	4.710001	0.808495	0.767076
smaple 1 b	3.946787	2.299416	0.511379	4.710001	0.808495	0.767076
sample 2 a	1.969755	1.338653	0.5491	1.925988	1.148832	0.564748
sample 2 b	1.969755	1.338653	0.5491	1.925988	1.148832	0.564748
sample 3 a	2.296423	0.918896	0.341982	1.855816	0.7011	0.368854
sample 3 b	2.296423	0.918896	0.341982	1.855816	0.7011	0.368854
sample 4 a	0.914766	0.354759	0.16443	1.02186	0.347879	0.105935
sample 4 b	0.914766	0.354759	0.16443	1.02186	0.347879	0.105935
COL1						
smaple 1 a	109.3324	19.5907	2.403751	36.31681	3.62826	2.298258
smaple 1 b	109.3324	19.5907	2.403751	36.31681	3.62826	2.298258
sample 2 a	16.94579	9.617305	2.791069	124.8827	24.51238	8.328088
sample 2 b	16.94579	9.617305	2.791069	124.8827	24.51238	8.328088
sample 3 a	16.5227	1.23068	1.435198	7.754564	1.535384	1.353736
sample 3 b	16.5227	1.23068	1.435198	7.754564	1.535384	1.353736
sample 4 a	9.839428	3.249153	1.077381	5.46191	1.539347	0.56219
sample 4 b	9.839428	3.249153	1.077381	5.46191	1.539347	0.56219
ALPL						
smaple 1 a	1.29329	0.428914	0.809686	0.755347	0.438557	0.239739
smaple 1 b	1.29329	0.428914	0.809686	0.755347	0.438557	0.239739
sample 2 a	2.909981	7.131557	2.850696	3.492574	8.956081	5.55502
sample 2 b	2.909981	7.131557	2.850696	3.492574	8.956081	5.55502
sample 3 a	14.73092	39.45602	10.45095	2.95076	3.296589	1.716117
sample 3 b	14.73092	39.45602	10.45095	2.95076	3.296589	1.716117
sample 4 a	2.395262	1.335632	1.048845	5.406691	9.731804	4.116244
sample 4 b	2.395262	1.335632	1.048845	5.406691	9.731804	4.116244

SPP1

smaple 1 a	1.163482	1.773022	1.618923	0.835627	1.182494	1.327075
smaple 1 b	1.163482	1.773022	1.618923	0.835627	1.182494	1.327075
sample 2 a	12.97434	8.153401	14.02873	4.029926	4.136408	3.571236
sample 2 b	12.97434	8.153401	14.02873	4.029926	4.136408	3.571236
sample 3 a	1.956433	0.807875	0.483648	1.139635	0.761415	0.704496
sample 3 b	1.956433	0.807875	0.483648	1.139635	0.761415	0.704496
sample 4 a	4.189182	1.745665	0.631599	4.279875	5.00001	3.473423
sample 4 b	4.189182	1.745665	0.631599	4.279875	5.00001	3.473423

BGLAP

smaple 1 a	0.352771	0.352771	6.883903	0.823181	0.823181	1819.067
smaple 1 b	0.352771	0.352771	6.883903	0.823181	0.823181	1819.067
sample 2 a	11.91874	5.62086	4604.534	1.853872	0.733341	1.765296
sample 2 b	11.91874	5.62086	4604.534	1.853872	0.733341	1.765296
sample 3 a	1.548459	0.375577	50.56972	1.01351	0.145103	1.99259
sample 3 b	1.548459	0.375577	50.56972	1.01351	0.145103	1.99259
sample 4 a	0.947996	0.374824	0.723532	0.59707	0.604249	6.595821
sample 4 b	0.947996	0.374824	0.723532	0.59707	0.604249	6.595821

SOX9

smaple 1 a	3.234397	14.56549	4.852911	149.4623	62.06094	62.44472
smaple 1 b	3.234397	14.56549	4.852911	149.4623	62.06094	62.44472
sample 2 a	7.440419	8.561685	8.817242	20.87562	22.75248	76.73141
sample 2 b	7.440419	8.561685	8.817242	20.87562	22.75248	76.73141
sample 3 a	16.66609	17.05381	15.39945	22.17871	23.94413	47.69914
sample 3 b	16.66609	17.05381	15.39945	22.17871	23.94413	47.69914
sample 4 a	4.455396	4.806917	13.44886	46.7514	66.94441	16.27719
sample 4 b	4.455396	4.806917	13.44886	46.7514	66.94441	16.27719

COL2

smaple 1 a	2.804423	0.467897	1.665396	48.07049	13088.22	2988.398
smaple 1 b	2.804423	0.467897	1.665396	48.07049	13088.22	2988.398
sample 2 a	30.12311	11.33625	21.24311	137.3781	75.73117	144.7292
sample 2 b	30.12311	11.33625	21.24311	137.3781	75.73117	144.7292
sample 3 a	404.4151	482.4644	1767.679	31.68907	157410.1	49289.35
sample 3 b	404.4151	482.4644	1767.679	31.68907	157410.1	49289.35
sample 4 a	796.4321	1999.13	42.95809	56.69841	179109.1	1645.904
sample 4 b	796.4321	1999.13	42.95809	56.69841	179109.1	1645.904

	ACAN					
smaple 1 a	1.146275	2.002927	9.93E-09	0.206901	0.432772	1.4E-08
smaple 1 b	1.146275	2.002927	9.93E-09	0.206901	0.432772	1.4E-08
sample 2 a	0.920855	0.663548	7.11E-08	1.63642	3.400114	1.27E-08
sample 2 b	0.920855	0.663548	7.11E-08	1.63642	3.400114	1.27E-08
sample 3 a	3.51479	1.724758	2.03E-08	1.819798	7.141355	1.81E-07
sample 3 b	3.51479	1.724758	2.03E-08	1.819798	7.141355	1.81E-07
sample 4 a	1.391807	11.05808	3.8E-08	4.447731	5.50308	2.71E-08
sample 4 b	1.391807	11.05808	3.8E-08	4.447731	5.50308	2.71E-08
