# Supplementary material for: Vertebral body versus iliac crest bone marrow as a source of multipotential stromal cells: Comparison of processing techniques, tri-lineage differentiation and application on a scaffold for spine fusion
Source: PLoS One. 2018 May 24;13(5):e0197969. doi: 10.1371/journal.pone.0197969 (PMC5967748; doi:10.1371/journal.pone.0197969)
Supplement: S5 File — (PDF) [file pone.0197969.s007.pdf]

% OF MSCs of CD45 NEGATIVE CELLS

	IC-BM	VB-BM
SAMPLE 1	71	51
SAMPLE 2	62	90
SAMPLE 3	68	61
Mean	67	67.33
Std. Error of M	2.646	11.7

ALP/DNA RATIO

	IC-BM E	IC-BM O	VB-BM E	VB-BM O
SAMPLE 1	0.003	0.1	0.008	0.23
SAMPLE 2	0.006	0.206	0.001	0.421
SAMPLE 3	0.007	0.044	0.06	0.135
SAMPLE 4	0.025	0.12	0.09	0.28
SAMPLE 5	0.02	0.054	0.02	0.076
SAMPLE 6	0.001	0.18	0.01	0.24
Mean	0.01033	0.1173	0.0315	0.2303
Std. Error of M	0.003997	0.02676	0.0145	0.04893
